# Short-chain fatty acids as a link between diet and cardiometabolic risk: a narrative review

**DOI:** 10.1186/s12944-023-01803-5

**Published:** 2023-03-14

**Authors:** Eline Birkeland, Sedegheh Gharagozlian, Jørgen Valeur, Anne-Marie Aas

**Affiliations:** 1 Section of Nutrition and Dietetics, Department of Clinical Service, Division of Medicine, Oslo University Hospital, Oslo, Norway; 2 Unger-Vetlesen Institute, Lovisenberg Diaconal Hospital, Oslo, Norway; 3 Institute of Clinical Medicine, University of Oslo, Oslo, Norway

**Keywords:** Diet, Short-chain fatty acids, Gut microbiota, Human studies, Metabolic effects

## Abstract

**Aim:**

Diet has a profound impact on cardiometabolic health outcomes such as obesity, blood glucose, blood lipids and blood pressure. In recent years, the gut microbiota has emerged as one of several potential key players explaining dietary effects on these outcomes. In this review we aim to summarise current knowledge of interaction between diet and gut microbiota focusing on the gut-derived microbial metabolites short-chain fatty acids and their role in modulating cardiometabolic risk.

**Findings:**

Many observational and interventional studies in humans have found that diets rich in fibre or supplemented with prebiotic fibres have a favourable effect on the gut microbiota composition, with increased diversity accompanied by enhancement in short-chain fatty acids and bacteria producing them. High-fat diets, particularly diets high in saturated fatty acids, have shown the opposite effect. Several recent studies indicate that the gut microbiota modulates metabolic responses to diet in, e.g., postprandial blood glucose and blood lipid levels. However, the metabolic responses to dietary interventions, seem to vary depending on individual traits such as age, sex, ethnicity, and existing gut microbiota, as well as genetics. Studies mainly in animal models and cell lines have shown possible pathways through which short-chain fatty acids may mediate these dietary effects on metabolic regulation. Human intervention studies appear to support the favourable effect of short-chain fatty acid in animal studies, but the effects may be modest and vary depending on which cofactors were taken into consideration.

**Conclusion:**

This is an expanding and active field of research that in the near future is likely to broaden our understanding of the role of the gut microbiota and short-chain fatty acids in modulating metabolic responses to diet. Nevertheless, the findings so far seem to support current dietary guidelines encouraging the intake of fibre rich plant–based foods and discouraging the intake of animal foods rich in saturated fatty acids.

## Introduction

The role of diet in health and chronic conditions such as obesity, insulin resistance, and cardiovascular disease is well known [1] and recognized in clinical guidelines [2, 3]. Diet also shapes the composition of the gut microbiota, which in recent years has emerged as one of several potential key players explaining dietary effects on health and disease [4, 5]. However, studying the relationship between health and microbiota in humans is difficult due to challenges of controlling for environmental factors in study subjects. Lately, a number of large-scale studies including more than 800 people have identified gut microbiota-diet interactions that associate with different cardiometabolic markers [5–7], but so far, only animal studies offer some evidence of causality. The human studies also reveal that the metabolic responses to food varies substantially partly due to individual differences in gut microbial composition and functions. Numerous studies also report that the gut microbiome of people with diseases, such as type 2 diabetes, stroke and immune-mediated inflammatory disease, is distinctly different from that of healthy individuals as they present with a microbiome with less diversity and reduced abundance of health promoting species [8–13].

Unravelling the interactions between diet and the gut microbiota and their impact on metabolism and cardiometabolic disease could open for new approaches to obtain good health and prevent and treat disease by feeding our gut microbiota the optimal diet. What the best diet is may differ from one individual to another depending on metabolic phenotype, existing microbiome and more [7, 14]. Identifying predictors of metabolic responses is another research field that needs to be mapped and tested in intervention trials. Finally, such research can be useful in the development of dietary guidelines and help optimize personalized diet recommendations based on prediction models derived from large studies of diet-microbiota interactions and effect on cardiometabolic health.

In this article, we review the current knowledge of diet and gut microbiota interaction focusing on the gut-derived microbial metabolites of short-chain fatty acids (SCFA) and their role in modulating cardiometabolic disease risk. Other dietary derived compounds produced by bacteria, such as trimethylamine (TMA), other methylamines, polyamines, and secondary bile acids may also affect host health, but is outside the scope of this narrative review.

## Method

In this narrative review, we did not perform a systematic search for articles, but publications were selected on a discretionary basis for their relevance to the aim of the review. We focused on studies in humans but included some studies in animal models and cell lines in cases where we found it relevant to underpin possible pathways of action, where these studies guided and supported findings in human studies and in the cases where human studies were lacking. Most included articles used were chosen from searches in Pubmed and Google scholar from May 2021 to July 2022 using the keywords: diet, short-chain fatty acids, gut microbiota, gut bacteria, cardiometabolic, prebiotics, SCFA receptors, LPS, inflammation, and combinations thereof. Additional publications were added using the snowballing method as a complementary approach, including relevant papers that were cited in already included publications and articles that came to our attention mentioned by colleagues or cited during a conference.

## Gut microbiota

**Fig. 1:**
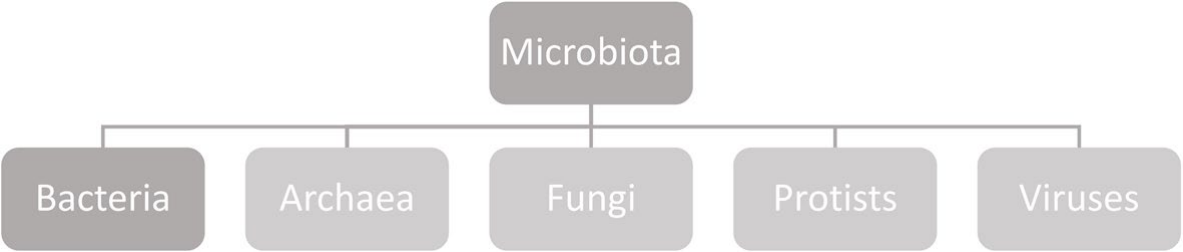
Overview of microbiota

**Fig. 2:**
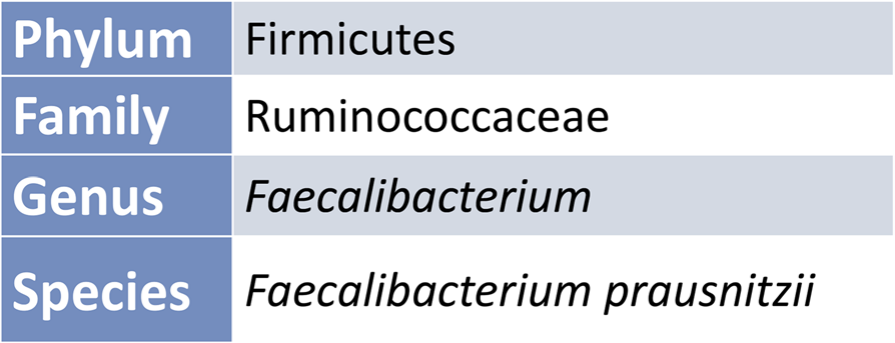
Simplified rank-based classification of the butyrate producer *Faecalibacterium prausnitzii*

**Fig. 3:**
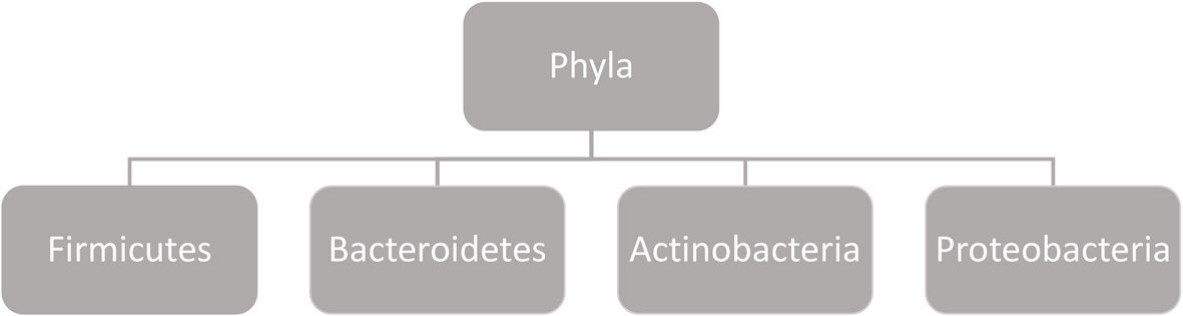
The major bacterial phyla in the gut

In clinical science microbiota as a term often describes bacteria. Although the bacteria account for the main mass, the microbiota comprises archea, protists, fungi and viruses as well (Fig. 1) [15]. The bacteria are ranked into phylum, order, class, family, genus and species (Fig. 2) [16]. The major bulk of bacteria in the gut are the phyla Firmicutes and Bacteroidetes (90%), followed by Actinobacteria and Proteobacteria (Fig. 3) [16]. Cyanobateria, Verrumicrobia, Tenericutes and other phyla not yet assigned, are included as well [16].

In 2004, a connection between gut microbiota and development of excessive body fat and insulin resistance was discovered in mice [17]. Further research on mice has reported increased capacity to extract energy from undigested food components and that obesity is transmissible between individuals through gut bacteria [18]. This was followed by a number of epidemiologic studies reporting differences in gut bacteria between healthy humans and humans with increased risk for cardiometabolic diseases [8, 9, 12, 19]. These differences include reduced microbial diversity, changed ratio of the two major phyla Firmicutes and Bacteroidetes, reduced concentrations of some species that are assumed healthy, and increased concentrations of others that are considered harmful [8, 9, 12, 19]. These differences are often called dysbiosis. Dysbiosis as a term, however, is disputable since it is unclear what constitutes a healthy core microbiota or whether it even exits [20]. So far, speculations about which traits frame a healthy bacterial composition in the gut, are deduced from the corresponding opposite features found between microbiota in healthy people and people with overweight and health problems. Nonetheless, these findings caused a spiked interest in gut microbiota as a future objective for management of cardiometabolic diseases.

### The size of the gut microbiota pool and changing perception of its role

Long ago, the quantity of bacterial cells in the human gut was estimated to largely exceed the number of human cells [21]. A recent recalculation, though, reduced the bacterial cell count to match the number of human cells [21]. Nevertheless, human beings have approximately 22 000 genes, whereas our bacteria all together have a hundred times more, with a far greater genetic capacity to produce and express biologically active compounds.

Our conception of the gut bacteria’s impact has altered from docile dwellers in the gut to an active bacterial society, with means to modulate the biology of their human hosts. This host–microbe relationship is believed to benefit human health, because the gut microbiota extracts nutrients from undigested dietary components, maintains the intestinal barrier, protects the host against harmful bacteria, produces essential vitamins, and modulates the immune system [22]. To what extent our gut inhabitants, with their outnumbering genes, are able to regulate human bodily functions, is a hot research field.

### What shapes the gut microbiota?

The bacterial composition changes throughout life and is affected by genetics, age, transit time through the gut, and a wide range of environmental factors, including mechanism of birth, breastfeeding, maternal microbiota, diet, lifestyle, medication, and state of health [22]. Knowledge about which factors that affect gut microbiota has increased considerably during the last years including factors that may have confounded earlier studies. The importance of controlling for diet, geographical residence, socio-economic status and medication when studying relations between microbiota and cardiometabolic diseases has since been emphasizes by several research groups [23–25]. Indeed, environmental factors appear to have even greater impact on the gut microbiota than genetics, and a recent study [26] found that as much as 20% of the variability between microbiota in people was associated with diet, medication and body composition [26]. The same authors estimated the overall microbiome heritability to be between 1.9% and 8.1%. Furthermore, the transmissibility of obesity between mice has also been shown to be diet-dependent and that a diet rich in fruits and vegetables and low in saturated fat appears to be protective [27].

### Gut microbiota composition, diversity, and function

Although the bacterial phyla stay relatively fixed in the healthy adult gut, the species themselves are highly susceptible [28]. Yet, the bacterial community is resilient, i.e., normally re-establishes after temporary disruptions of diet or medication [28]. The microbial diversity can be defined as “the number and abundance distribution of distinct types of organisms” and high diversity is associated with good health [29]. The diversity has been reported to increase during childhood, remain stable in adulthood and decline in old age [30], although a study by Odamaki et al*.* suggests that the observed decline in elderly people may be confounded by external factors such as residing in own home or in an institution [31].

The gut bacteria have various capabilities and perform different tasks in the colon [29, 32, 33], but different species may possess common abilities. This means that healthy individuals may have dissimilar compositions of well-functioning bacterial communities. Even if the composition of gut microbiota varies considerably between people at species level, the bacterial functions appear to vary less between people than the actual species [29, 33]. A healthy core of microbial functions may thus turn out to be more relevant than a healthy core microbiota composition.

### Dietary fibres are fermented by the gut microbiota to SCFA

Carbohydrates that humans are unable to digest themselves are referred to as dietary fibres. They pass through the small intestine into the colon where they are fermented by the microbiota [34]. The indigestible but fermentable dietary fibres from plant-based food are the preferred energy source for the gut bacteria [35], which ferment the fibres into various compounds, including SCFA [35].

The fermentation process of fibres from plant-based food increases the luminal acidity in the colon, which provides an environment more suited to healthy bacteria than harmful bacteria [36]. The gut bacteria are also capable of fermenting proteins, but appear to do so only if the fibres from plant-based food are short in supply [36].

Prebiotic fibres are defined as; substrates selectively utilised by host microorganisms conferring a health benefit [37]. During the recent years they have been highlighted as a possible treatment approach in overweight and cardiometabolic diseases [38–43]. Galacto-oligosaccharides (GOS) and inulin-type fructans (ITF) are among the most investigated prebiotics. Clinical trials report beneficial effects on glycaemic regulation, suppression of energy intake and appetite as well as weight loss after treatment with prebiotic fibres, including ITF and GOS [37, 38, 41–46].

### Does the microbiota modulate postprandial responses to diet?

Diet plays a major role in shaping the gut microbiota, but does the gut microbiota modulate the postprandial responses to food intake, and can this explain the heterogeneity in metabolic effects of dietary interventions? In 2015, Zeevi et al*.* [7] monitored glucose levels continuously for a week in 800 healthy and prediabetic individuals, and measured responses to over 46 000 meals. They found that the response to identical meals were highly variable. A machine-learning algorithm was made based on the measured blood parameters, dietary habits, anthropometrics, physical activity, and gut microbiota in this cohort. The algorithm accurately predicted individual postprandial glycaemic responses to typical meals eaten during the intervention. The authors validated these predictions in a new cohort and performed a randomised controlled trial using this algorithm. A dietary intervention based on the predictions lowered postprandial responses significantly and resulted in consistent changes in gut microbiota composition [7].

More recently, the PREDICT study, with over 1000 men and women, showed that individual factors, like the gut-microbiota, had more impact than macronutrient composition on postprandial lipidemia (7,1% of variance compared with 3,6%), but not postprandial glycaemia (6% vs 15,4%) [14]. The authors validated their findings in an independent cohort and devised a machine-learning model that predicted both triglyceride and glycaemic response to food intake. Interestingly, genetic variations only partially influenced the predictions (9.5% for glucose, 0.8% for triglycerides, and 0.2% for C-peptide) [14]. In the PREDICT study, deep metagenomics sequencing of 1203 gut microbiomes derived from faecal samples from the participants was also performed. Several associations between gut microbes and specific nutrients, foods, food groups and general dietary indices were found, which were driven by the presence and diversity of healthy and plant-based foods [5]. Overall microbiome composition was predictive for many cardiometabolic blood markers including fasting and postprandial glycaemic, lipidemic and inflammatory indices. Microbiome signatures grouped both microbiome and dietary components into health-associated clusters that were in agreement with dietary quality and diversity scores.

A Dutch study showed that dietary patterns derived from cluster analyses of food frequency questionnaires (FFQs), associated with pro-inflammatory and anti-inflammatory features of the gut microbiome [6]. Shotgun metagenomics sequencing was performed in faecal samples from 1425 individuals to investigate gut microbial composition and function. Processed foods and animal-derived foods were consistently associated with higher abundances of Firmicutes, *Ruminococcus* species of the *Blautia* genus and endotoxin synthesis pathways. On the other hand, plant foods and fish correlated positively with SCFA-producing bacteria and pathways of nutrient metabolism. Gut bacteria known for their shared function in health and disease were consistently associated with the identified dietary patterns. In addition, specific foods and nutrients correlated with bacterial species that are known to have anti-inflammatory effects and protective effects on the gut mucosa. These diet–gut microbiome associations were found both in patients with intestinal disease and the general population [6].

## Effect of diet on gut microbiota and cardiometabolic risk factors

It is primarily fibres from plant-based foods that can be degraded to SCFA by intestinal bacteria. Mice fed a diet without soluble fibre developed inflammation in the gut and poor intestinal health, which in turn led to weight gain [47]. Intestinal health was restored after soluble fibre was reintroduced into the diet. Several mice studies also show that high-fat diets, in particular high-fat diets rich in long-chain saturated fatty acids (SFA), are linked to unfavourable changes in type and numbers of gut bacteria, resulting in dysbiosis and inflammation, with a subsequent increased risk of chronic disease, such as obesity and metabolic syndrome [48–51]. Dietary intervention trials in humans suggest that the microbiota-mediated effect of a dietary change on metabolism and health may be modest [52–55].

### Human studies with high-fat diets

In people who ate a very low-carbohydrate diet which contained little carbohydrate (4 E %) and fibre and correspondingly more fat (61 E %) and protein (35 E %), a lower bacterial colonization in the colon has been shown [56]. Faecal butyrate concentrations and abundance of the *Roseburia*/*E. rectale* group are both reduced in people eating a carbohydrate-reduced diet (24 g/day) [57]. In a randomised controlled-feeding trial Wan et al*.* [52] compared three dietary patterns differing in carbohydrate and fat proportions: a lower-fat diet (fat 20% energy), a moderate-fat diet (fat 30% energy) and a higher-fat diet (fat 40% energy). The study was performed among 217 young, healthy adults during a 6 month’s period. The researchers showed that the high-fat diet had unfavourable effects on the gut microbiota, faecal bacterial metabolites, and markers of inflammation, whereas the lower-fat diet was associated with a more favourable profile of these biomarkers.

In a recent systematic review, both interventional and observational studies showed associations between high fat diet intake, mainly rich in SFA, and reduction of bacterial abundance, diversity, and richness in the gut [4]. The dietary intervention studies showed no strong effects on gut microbiota and no association with metabolic outcomes. However, in observational studies high intake of total fat was positively correlated with the abundance of *Clostridium bolteae* and circulating serum levels of SFA correlated with *Blautia*, and both bacteria associated with unhealthy metabolic outcomes, i.e., insulin resistance and higher BMI or waist circumference [4]. Results from studies on diets rich in monounsaturated fatty acids (MUFA) were less consistent with no or possibly negative effects on total bacterial numbers and gut microbiota richness and diversity. In contrast, diets rich in omega-3 or omega-6 polyunsaturated fatty acids (PUFA) did not seem to affect the gut microbiota or metabolic health outcomes negatively. PUFA-enriched diets were associated with increased abundance of the Tenericutes phylum which in turn was associated with lower levels of triglycerides in plasma [4].

Evidence from randomised trials assessing the effect of PUFA on human gut microbiota is scarce. In a randomised cross-over trial Watson et al*.* [58] showed that a daily intake of 4 g omega-3 PUFA supplement (administered in capsules or drinks) over 8 weeks in 22 middle-aged, healthy volunteers was associated with reversible changes at gut family and genus levels, including an increase in the SCFA producing *Bifidobacterium*, *Lactobacillus*, *Lachnospira* and *Roseburia*. The authors concluded that the increase in density of butyrate producers was in line with existing preclinical literature and compatible with the known anti-inflammatory properties of omega-3 PUFA.

### Human studies with high-fibre diets

Intervention trials in humans with fibre supplements or fibre enriched diets have consistently shown a positive effect on gut microbiota composition, with an increase in SCFA-producing bacteria and SCFA in faeces or blood samples [59–63]. Wheat bran supplementation (> 70% arabinoxylan oligo-saccharides) increased the abundance of butyrate, acetate, and propionate as well as total SCFA concentrations in a human intervention trial [61]. However, increased faecal bulking and reduced transit time seen with increased dietary fibre, would decrease colonic absorption of SCFA and could partly explain the increases in faecal SCFA concentrations observed in studies with increased dietary fibre content [64].

A systematic review and meta-analysis of the effect of dietary interventions with fibre (mainly supplements) on gut microbiota in people with type 2 diabetes, showed that dietary fibre improved the relative abundance of *Bifidobacterium* and total SCFA, and improved glycated haemoglobin [65]. This systematic review included our own intervention study with 16 g per day of ITF for 6 weeks which induced moderate alterations in the composition of faecal bacteria, with an increased concentration of bifidobacteria being the most pronounced effect [66]. Compared to placebo, the prebiotic treatment also increased faecal concentrations of total SCFA, acetate, and propionate, but did not positively affect butyrate or the overall bacterial diversity [66]. Furthermore, the prebiotics had no positive effect on concentrations of glucose, insulin, gut hormones (GLP-1, GLP-2, PYY and ghrelin), appetite ratings or energy intake [67, 68].

Evidence from a recent systematic review suggests that ITF have a prebiotic effect on the gut microbiota, promoting the abundances of *Bifidobacterium, Lactobacillus,* and *Faecalibacterium prausnitzii* [69]. Beneficial health effects reported after intake of ITF included improved intestinal barrier function and laxation, increased insulin sensitivity, improved lipid profile, increased absorption of calcium and magnesium, and increased satiety [69]. However, another recent systematic review of ITF interventions in humans observed favourable effects of ITF intake on blood glucose, total cholesterol, and triglyceride concentration only in subjects with prediabetes and diabetes [70].

Three days with an evening meal of barley-kernel based bread rich in β -glucan fibres, increased fermentation activity and serum levels of SCFA in healthy adults, resulting in increased levels of gut hormones involved in regulation of blood glucose and appetite (GLP-1, PYY, and GLP-2), as well as improved insulin sensitivity [71]. Another study showed that a supplement with 3 g/d high molecular weight β-glucan increased Bacteroidetes and decreased Firmicutes abundances compared to placebo. At the genus level, the β-glucan supplement increased *Bacteroides*, and tended to increase *Prevotella* while decreasing *Dorea* [72]. However, low molecular weight β-glucan did not alter the intestinal microbiota composition [72]. The abundance of *Bacteroides, Prevotella*, and *Dorea* correlated with changes in risk factors for cardiovascular disease, such as BMI, waist circumference, blood pressure, and triglyceride levels [72]. This indicates that high molecular weight β-glucan can induce shifts in the intestinal microbiota that may partly explain the beneficial effect of β-glucan fibres on metabolism [72].

### SCFA and effects on metabolism

#### SCFA production, uptake, and turnover

Many of the beneficial health effects of dietary fibre, including prebiotic fibres are believed to be mediated through the microbial production of SCFA [73–76]. The SCFA mainly comprise acetate, propionate and butyrate, but include formate and lactate as well [75]. The molar ratio of acetate, propionate and butyrate in faeces is approximately 60:20:20 [77]. The bacteria can also metabolise lactate into acetate, propionate and butyrate [75].

SCFA are readily absorbed at similar rates in different parts of the colon [78]. They are then metabolized at three major sites in the body: ceco-colonic epithelium, liver cells and muscle cells [79]. Butyrate is the major energy source for ceco-colonic epithelium for maintenance-energy producing pathways [80]. Propionate is mainly used for gluconeogenesis in the liver, together with butyrate [81]. Acetate is also largely taken up by the liver. It enters the peripheral circulation to be metabolized by peripheral tissues, where oxidation of residual acetate is used for energy in muscle cells [82, 83]. Propionate and acetate appear less studied than butyrate, but may in addition to butyrate have anti-carcinogenic properties, and propionate is suggested to reduce visceral fat and liver fat [75].

SCFA act on host physiology through G protein-coupled receptors (GPCRs) and post-translational modifications [84, 85]. GPCRs are the largest receptor family in mammals, where six of them are sensitive to SCFA; GPR41 (also called free fatty acid receptor 3/FFAR3), GPR42, GPR43 (FFAR2), GPR109, GPR164, and olfactory receptor 78 (Olfr78) [86, 87]. These receptors are found in intestinal epithelium, immune cells, and fat cells, but their level of expression varies between tissues and cell types [85], and activation of the GPCRs may induce different effects in various tissues [88].

#### SCFA and inflammation

A large body of evidence suggests that an abnormal amount of lipids, hyperglycaemia and impaired insulin sensitivity can all cause endothelial dysfunction and low grade chronic inflammation, which have been linked to cardiovascular disease [13, 89, 90]. SCFA has a very potent anti-inflammatory effect, which block liberation of inflammatory mediators and, thus, reduces influx of immune cells to the site of inflammation, migration of immune cells, proliferation and persuades apoptosis [85, 91]. In this way functions mediated by GPCRs activation regulate the inflammatory process by preventing white blood cells from passing through the endothelium [92]. Furthermore, butyrate and acetate has been shown to exert beneficial effects on Angiotensin II-induced endothelial dysfunction in mice, by increasing the bioaccessibility of Nitric oxide (NO) and, thereby, reducing oxidative stress [93]*.* This effect was associated with GPR activation [93].

SCFA have effects on intestinal permeability and may, thus, regulate the systemic exposure to pro-inflammatory bacterial products, such as endotoxins or lipopolysaccharides (LPS) [94]. LPS are an essential part of the cell surface of most gram-negative bacteria [95, 96]. Translocation of LPS into the bloodstream induce development of low-grade endotoxaemia mostly by Toll-like receptor 4 (TLR4), acting as a receptor for LPS, signalling on macrophages, monocytes, and other cells of the inborn immune system. Furthermore, cluster of differentiation 14 (CD14) plays an important role in passing LPS to the TLR4 complex [97].

Patients with type 2 diabetes, overweight and atherosclerosis have increased levels of inflammatory cytokines, such as tumor necrosis factor alpha (TNF-α) and interleucin-6 (IL-6) [98–101], and LPS, which is caused by low-grade endotoxaemia [97, 102, 103].

High-fat diets have been shown in studies with humans and mice to increase the degree of LPS-containing microbiota in the intestine [104–107]. Increased blood levels of LPS (endotoxaemia) are probably linked to changes in the intestinal microbiota, since antibiotic therapy reduces caecal and systemic LPS levels simultaneously with reduced glucose intolerance and fat mass development [97, 107, 108].

In an experiment in rats, a diet rich in fat and cholesterol enhanced the level of propionate simultaneously with a decline in butyrate, compared to a control group that received standard food for laboratory rats [109]. The reduction in butyrate with time was linked to a raise in LPS liberated in the blood.

#### SCFA and regulation of appetite and blood glucose

**Fig. 4:**
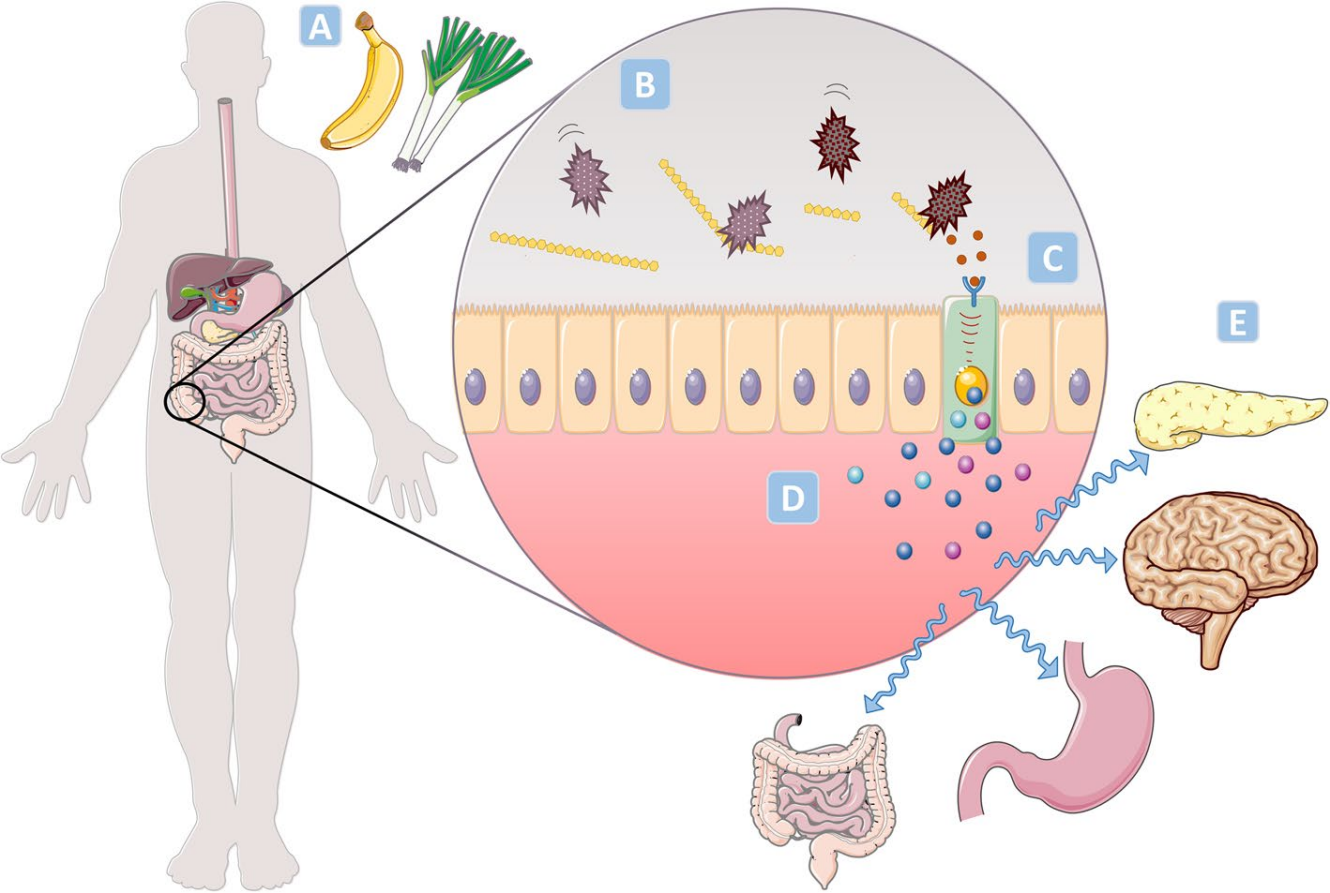
Effect of dietary fibre on glycaemic control and appetite, and possible pathways. **A** Dietary fibres escape digestion in the small intestine and **B**) are fermented into SCFA acids by gut bacteria in the colon. **C**) The SCFA bind to G-protein coupled receptors in enteroendocrine L-cells. **D** This causes increased secretion of GLP-1, GLP-2, leptin and PYY in response to a meal. E) GLP-1 improves regulation of blood glucose by enhancing release of insulin and suppressing release of glucagon from pancreas. GLP-1 also protects the beta-cells. GLP-1, leptin, and PYY enhance satiety by affecting the brain and the gastrointestinal system. GLP-2 maintains the intestinal barrier and may, thus, prevent systemic inflammation. GLP-1 and 2, glucagon-like peptide 1 and 2; PYY, peptide YY; SCFA, short-chain fatty acids. *Figure was produced using Servier Medical Art and reproduced from first author’s thesis*

SCFA have been shown to bind to GPR41 and GPR43 in enteroendocrine L-cells and thereby increase release of the gut hormones peptide YY (PYY), glucagon-like peptide-1 (GLP-1) and glucagon-like peptide-2 (GLP- 2) after a meal (Fig. 4) [110]. This has the potential to improve glycaemic control and appetite regulation, as GLP-1 increases insulin release from pancreas and both GLP-1 and PYY are appetite suppressing hormones [111]. GLP-2 is important for preserving intestinal integrity [111, 112]. Animal studies show that butyrate improves glucose control by promoting gut production of GLP-1 and PYY as well as protecting the intestinal barrier [77, 113–115]. According to Sakakibara et al. [116], SCFA can also increase secretion of the appetite supressing hormone leptin by activating GPR43, based on in vivo and in vitro studies.

#### SCFA and lipid metabolism

The SCFA can enter the circulation as substrates for lipid and cholesterol synthesis in the liver but can also be a regulatory factor in lipid metabolism [84]. SCFA can enhance fatty acid oxidation and production of heat, block fatty acid synthesis, and reduce storage of fat in the body [117]. Angiopoietinlike 4 (ANGPTL4) is a signalling protein with several different functions that is synthesized in most tissues [118]. Studies suggest that ANGPTL4 is a key host protein that is reactive to the intestinal microbial environment. By controlling fatty acid uptake and metabolism in the tissues, ANGPTL4 can modify obesity in humans [119, 120]. SCFA, especially butyrate, affects lipid metabolism by inducing secretion of ANGPTL4 in human colon cell lines, which stimulates peroxisome proliferator-activated receptor-γ (PPARγ) and, thereby, blocks the activation of lipoprotein lipase (LPL) [119, 121]. LPL is important for the transfer of fatty acids from chylomicrons and very-low-density lipoprotein (VLDL) to adipocytes [122]. With low LPL activity, less fat is stored in adipose tissue [104, 123], and triglyceride-rich lipoproteins remain in the blood stream for a longer period, raising fat accumulation in and on the artery walls which may lead to atherosclerosis [124]. Furthermore, the excessive expression of ANGPTL4 in white adipose tissue decreases fat mass [125].

Finally, butyrate increases heat production and lipids utilisation through the uncoupling protein (UCP), which performs an essential role in lipid metabolism, and also improves lipid metabolism by activating adiponectin [84].

However, the role of SCFA in lipid metabolism and obesity remains controversial. Turnbaugh et al*.* [18], demonstrated in 2006 that SCFA can result in additional weight gain due to contribution of extra calories in the obese mice. Perry et al*.* [126] also indicated in 2016 that increased acetate turnover led to increased obesity and impaired insulin sensitivity in rodents.

#### SCFA and blood pressure regulation

The three main SCFA made by the microbiota in the intestinal lumen regulate blood pressure through Olfr78 and GPR41 [127, 128]. Olfr78 knock-out mice are hypotensive [129], whereas GPR41 knock-out mice are hypertensive [130], suggesting that these pathways may be important in linking SCFA and host blood pressure control. Acetate and propionate operate via a complex interaction that results in renin secretion mediated through Olfr78 and counter-regulation through GPR41. Butyrate works via attenuation of angiotensin II-induced expression of renal prorenin receptors and renin [76]. Olfr78 is expressed at high rates in the renal juxtaglomerular apparatus, where it causes increased renin secretion in response to SCFA binding [129]. Furthermore, both Olfr78 and GPR41 are expressed in smooth muscle cells of small resistance vessels, where they differentially mediate vascular tone [129].

#### Human studies with SCFA supplementation

Human studies with direct supplementation with SCFA are limited but largely in line with findings from animal models and cell-lines that support a beneficial role of SCFA in regulation of body weight, appetite, and energy expenditure as well as glycaemic control and insulin sensitivity [59]. In a study by Chambers et al*.* [131], propionate supplementation seemed to protect against weight gain when it was given to people as part of a habitual diet. Three other studies in humans have also shown that supplementation with propionate, targeting delivery in the lower gastrointestinal tract, may reduce energy intake [131–133]. Furthermore, human studies also support the observation in rodents that SCFA stimulate whole-body lipid oxidation and thereby increases energy expenditure [134–136]. Blaak et al*.* [59] summarise the findings from these and other studies in a recent review, where they conclude that SCFA administration studies and dietary intervention studies with prebiotics with the aim to increase SCFA production in humans, provides direct and indirect evidence for a beneficial effect of SCFA on blood glucose regulation and insulin sensitivity. Nevertheless, this has not been shown in all recent well-controlled studies, and Blaak et al*.* [59] mention that the lack of effect is mostly observed in metabolically disturbed phenotypes, which suggests a disturbed SCFA handling/signalling in these individuals [59]. Interestingly, this is in accordance with our findings of no effect of ITF on glycaemic control and appetite regulation in patients with type 2 diabetes [67, 68], but is in contrast to the findings of Liu et al*.* [70] mentioned earlier, who found that interventions with ITF only had favourable effects on metabolic outcomes in people with prediabetes and diabetes.

## Conclusions and future perspectives

In this narrative review, we have summarised research supporting the important role of diet in shaping gut microbiota and the intestinal production of SCFA. SCFA seem to have a significant impact on metabolic regulation and can, therefore, modulate cardiometabolic risk. This research field is still relatively young, so robust conclusions about causality cannot be made. Even though large methodological advances have been made, allowing us to investigate both the composition and the functionalities of the gut microbiota, limitations still exist in studying interaction between diet and gut microbiota in humans. Animal and cell line studies have paved the way for hypotheses to be explored in human studies, and the limited evidence we have from human studies so far, seem mainly to support the beneficial role of SCFA in regulation of blood glucose, blood lipids, and energy homeostasis. However, the underlying mechanisms for SCFA effects are still just being uncovered, and these studies are difficult to replicate in humans.

To broaden our understanding of diet-microbiota interaction in cardiometabolic health, and the role of SCFA in this interplay, several knowledge gaps have to be filled and methodology improved: First, we need better and more detailed and precise dietary investigation methods. The current investigation methods, like FFQs, are inherently imprecise. The use of dietary records, biomarkers of dietary intake and standardized meal challenges can complement information from FFQs. It is important to look beyond nutrients and single foods in diet–microbiome research, and one way of achieving a better understanding of this interaction is to perform unsupervised clustering analysis to identify dietary patterns and microbial clusters, like they did in the study by Bolte et al*.* [6].

Second, we should strive to develop more detailed and precise methods to identify and classify microbiota and SCFA in the human gut. Today’s analyses in human studies are mainly restricted to faecal analyses whereas the main part of the SCFA is absorbed and used as substrate in the colonocytes. As just a minor percentage of the SCFA produced in the gut are excreted in the faeces, faecal analyses of SCFA may give a false impression of dietary interventions’ effect on gut microbiota and SCFA production.

Finally, we need a better understanding of the relationship between gut bacteria and human health. In the PREDICT study, the strongest microbiome–habitual diet associations were driven by poorly characterized microorganisms, which strongly suggests that our knowledge of the bacteria and their metabolic functions, is still far from complete and needs further investigation [5]. Future investigations of the remaining microbes that accompany the bacteria in the gut may contribute to higher understanding of human health and means to conquer illness. Further research, especially large, well‐powered, long‐term human intervention studies, is required to further understand and promote the role diet plays in modulating the gut microbiota and SCFA production and, thereby, human metabolism and health. These studies should be performed in populations that differs both geographically and with regard to sex, age, metabolic phenotypes, and health.

This narrative review gives an updated overview of a rapidly evolving research field that includes many different research disciplines and tries to connect them to yield a deeper understanding. At the same time, such a multidisciplinary approach may overlook details pertaining to the individual disciplines. Importantly, the clinical bearing of experimental basic studies must eventually be tested in well-designed clinical trials. The greater part of the research in this field is in-vitro and animal-studies. We chose to focus on human studies in this review and may thus have left out other types of studies of interest. Many of the human intervention studies included are acute studies and most of the long-term studies had a relative short duration of three to 12 weeks and a limited number of participants (*n* = 6–30) [4, 59, 63]. Our findings are also limited by the fact that the literature was gathered in a non-systematic way which may have caused some selection bias, as well as the fact that we did not formally analyse risk of bias in the intervention studies included.

What studies have found to promote diversity, increase SCFA production and be associated with beneficial effects on cardiometabolic risk factors, seems to be in line with the general guidelines for a healthy diet with an emphasize on plant-based foods like vegetables, fruit, berries, whole grains, nuts and fish. Furthermore, an unfavourable cardiometabolic profile is associated with highly processed foods rich in sugars, refined carbohydrates, fat, and saturated fat in particular. However, the metabolic responses to dietary interventions seem to vary depending on individual traits such as sex and existing gut microbiota, as well as genetics. With increasing knowledge about factors shaping the gut microbiota, the potential to develop personalized dietary recommendations in prevention and treatment of disease and metabolic disturbances seems promising.

## Data Availability

Not applicable.

## Abbreviations

ANGPTL4: Angiopoietin-like 4
FFAR: Free fatty acid receptor
CD14: Cluster of differentiation 14
FFQ: Food frequency questionnaire
GLP-1: Glucagon-like peptide-1
GLP-2: Glucagon-like peptide-2
GOS: Galacto-oligosaccharides
GPCR: G protein-coupled receptor
IL-6: Interleukin 6
ITF: Inulin-type fructans
LPL: Lipoprotein lipase
LPS: Lipopolysaccharides
MUFA: Monounsaturated fatty acids
Olfr78: Olfactory receptor 78
PYY: Peptide YY
PUFA: Polyunsaturated fatty acids
SCFA: Short-chain fatty acids
SFA: Saturated fatty acids
TLR: Toll-like receptor
TNF-α: Tumor necrosis factor alpha
TMA: Trimethylamine
UCP: Uncoupling protein
